# Efficacy and safety of hypomethylating agents in the treatment of AML/MDS patients relapsed post allogenetic hematopoietic stem cell transplantation

**DOI:** 10.3389/fonc.2024.1465334

**Published:** 2024-12-09

**Authors:** Yaxin Wang, Qingyun Wang, Hanyun Ren, Yujun Dong, Qingya Wang, Zeyin Liang, Yue Yin, Wei Liu, Weilin Xu, Na Han, Yuan Li

**Affiliations:** Department of Hematology, Peking University First Hospital, Beijing, China

**Keywords:** hypomethylating agents, venetoclax, myeloid malignancy, allo-HSCT, relapse

## Abstract

**Introduction:**

Acute myeloid leukemia (AML) and myelodysplastic syndrome (MDS) constitute myeloid malignancies, and allogeneic hematopoietic stem cell transplantation (allo-HSCT) is considered as a potentially optimal approach for achieving a long term cure. However, post-allo-HSCT relapse remains a leading cause of mortality and therapeutic failure.

**Methods:**

To evaluate the efficacy and safety of combining hypomethylating agents (HMAs) with Bcl-2 inhibitors in the treatment of AML/MDS relapse following allo-HSCT, we retrospectively collected data from 42 patients who experienced relapse between April 2012 and March 2022 at Peking University First Hospital. Among these patients, 21 underwent intensive chemotherapy (IC) alone, while the other 21 received treatment with HMAs after IC treatment, either alone or in combination with the Bcl-2 inhibitor venetoclax (VEN).

**Results:**

The median overall survival (OS) was 9 ± 2.153 months, and the one-year OS rate was 41.5%. The overall response rate (ORR) in the chemotherapy group and the IC+HMAs ± VEN group was 52.38% (11/21) and 76.19% (16/21), respectively, with no significant difference found (P=0.107). Kaplan-Meier analysis revealed a significant difference in OS between the chemotherapy group and the IC+HMAs ± VEN group in our retrospective cohort study (P=0.041, χ2= 4.016). Additionally, a significant difference in overall survival (OS) rates was observed between the two groups for patients categorized as intermediate/high risk (P=0.008). The secondary relapse rate was 45.45% (5/11) in the IC cohort and 25% (4/16) in the IC+HMAs ± VEN group, respectively, with no significant difference identified between the two cohorts (P=0.268). Furthermore, upon assessing the risk of graft-versus-host disease (GvHD), infection, and agranulocytosis, no notable differences were observed with the use of HMAs, suggesting that HMAs did not increase the risk. In the IC+HMAs ± VEN group, 7 patients received VEN in addition to HMAs, and no significant statistical difference was found in OS when comparing patients who received HMAs alone and those who received HMA+VEN (P=0.183), also, a statistically significant difference in OS was noted between the two groups whenaccounting for competing risks (P=0.028).

**Conclusions:**

This retrospective study highlights the efficacy of IC+HMAs ± VEN in treating AML/MDS patients experiencing relapse post allo-HSCT, improving survival rates, especially for those classified as intermediate/high risk, with favorable tolerability.

## Highlights

- Relapse post allo-HSCT results in poor outcome.
- HMAs ± VEN enhance the effectiveness without increasing the risk of adverse events.
- VEN concentration must be monitored during treatment to minimize the risk of adverse effects.

## 1 Introduction

To summarize, acute myeloid leukemia (AML) and myelodysplastic syndromes (MDS) are hematologic malignancies with a poor prognosis. Although allogeneic hematopoietic stem cell transplantation (allo-HSCT) has improved the prognosis for many patients, relapse remains a significant issue, with a 22%-55% occurrence rate post-transplantation (1), making it the leading cause of death (2, 3), The 2-year survival rate is only 20% (1). Studies suggest that relapse may be related to immunological escape, which could be due to evasion from T-cell alloreactivity and the dysregulation of pro-inflammatory and anti-inflammatory factors. Based on these mechanisms, salvage therapies such as intensive chemotherapy alone (IC), donor lymphocyte infusion (DLI), and secondary HSCT have been used to treat patients with post-transplant relapse, but their efficacy and safety are dismal. According to a retrospective analysis from the European Society for Blood and Marrow Transplantation (EBMT), the 2-year-OS rate for patients treated with DLI after their first relapse post-HSCT was 21%, signifying a significant difference compared to those who did not receive DLI (2-year-OS 9%, P <0.001) (4). Yalniz et al. (5) reported a 5-year OS rate of 32% for patients undergoing secondary HSCT. Newer treatment options, including CAR-T cell therapy, small molecular inhibitors (e.g., FLT-3 inhibitors and IDH1/2 inhibitors), and antibodies, are emerging for the treatment of AML and MDS relapse after allo-HSCT, holding promise for improved outcomes in the future (6, 7).

Multiple studies have demonstrated that hypomethylating agents (HMAs) can effectively reduce DNA methylation levels in tumor cells, reactivating silenced tumor suppressor genes and promoting the apoptosis of senescent or malignant cells (8). HMAs have been demonstrated to postpone relapse in AML patients achieving complete remission (CR) or complete remission with incomplete blood count recovery (CRi) through induced chemotherapy, leading to extended overall survival (OS) and disease-free survival (DFS) (9). Furthermore, HMAs may serve as a valuable maintenance therapy in patients following hematopoietic stem cell transplantation (HSCT), contributing to prolonged disease remission (8). In patients with relapsed/refractory (R/R) MDS/AML, HMAs can induce a graft-versus-leukemia (GvL) effect without elevating the risk of GvHD. CD4+T cells, particularly regulatory T cells (Tregs), play a pivotal role in establishing and maintaining tolerance post-HSCT. *In vitro* studies have indicated that HMAs may up-regulate the expression of FoxP3, essential for Tregs expansion (10). In a phase-II single-center clinical trial conducted by Sung Won Choi, when treated with HMAs, the incidence of grade II-IV graft-versus-host disease (GVHD) 100 days after unrelated donor HSCT was 25%, with a rate of grade III-IV GVHD at 8%, both of which were lower compared to the use of tacrolimus/methotrexate as a GVHD prevention strategy (11). This suggests the potential for an enhanced prognosis in patients with relapsed MDS or AML post-transplantation (12).

The BCL-2 family plays a pivotal role in regulating apoptosis in aging cells. It comprises anti-apoptotic molecules like Bcl-2, Bcl-xL, Bcl-w, A1/Bfl-1, and others. Furthermore, pro-apoptotic molecules, including Bak, Baf, and others, are integral components of this family. The Bcl-2 family predominantly governs apoptosis by regulating the release of Cytochrome-c from mitochondria and also modulates cell cycle apoptosis. VEN, a small molecule BCL-2 inhibitor, binds to Bcl-2, suppressing its anti-apoptotic effect and facilitating the activity of pro-apoptotic molecules (13, 14). Recent studies suggest that combining VEN with HMAs can improve the prognosis of high-risk AML or MDS by directly inhibiting leukemia and preventing GvHD. Furthermore, accumulating evidence suggests that the combination of VEN and HMAs shows promise in treating and preventing relapsed AML or high-risk MDS post hematopoietic stem cell transplantation (HSCT) (15–17). However, these studies had limitations concerning the number of cases, age, risk classification, and further research is warranted.

To assess the effectiveness and safety of HMAs and VEN in patients who experienced a relapse after undergoing allo-HSCT, we conducted a retrospective cohort study involving 42 patients treated at our center.

## 2 Methods

### 2.1 Patients

We conducted a retrospective study at Peking University First Hospital from April 2012 to March 2022, collecting 42 patients who underwent allo-HSCT and subsequently experienced relapse, specifically for AML or MDS. Among these patients, 21 received treatment with HMAs ± VEN. Simultaneously, the remaining 21 patients, who exclusively underwent intensive chemotherapy (IC), were chosen as the control group. The control group was paired with the patients receiving HMAs ± VEN in a 1:1 ratio, considering age, gender, disease, cytogenetics, extramedullary relapse, and time to relapse. AML classification adhered to the 2022 European Leukemia Net (ELN) recommendations (18), High-risk MDS was determined based on World Health Organization (WHO) Prognostic Scoring System (WPSS) points ≥ 3 or International Prognostic Scoring System-Revised (IPSS-R) scores > 4.5. The collected patients comprised 26 males and 16 females, with a median age of 41.5 years. The median time from HSCT to relapse was 7 months, and the median follow-up period extended to 8.5 months. All collected patients exhibited normal liver and kidney function, and their Eastern Cooperative Oncology Group (ECOG) score was ≤ 2 points. The study received approval from the Review Board of Peking University First Hospital (2023 Research 291-001) and was conducted in strict adherence to the Declaration of Helsinki.

### 2.2 Treatment modalities

In this study, patients were allocated to two groups: the IC group and the IC+HMA ± VEN group. In the IC group, twenty-one patients underwent 1 to 7 cycles of CLAG (Cladribine, Ara-C, G-CSF), HAA (homo harringtonine, Ara-C, Acla/DNR), or MAE (mitoxantrone, Ara-c, VP-16) regimens, with a median of 3 cycles, our decision to option for an intensive chemotherapy regimen is contingent upon the patient’s overall health condition and their previous treatment history. In the IC+HMA ± VEN group, eighteen patients experienced hematological relapse. The 18 patients initially underwent 1-2 cycles of IC to reduce tumor burden, and subsequently received HMAs ± VEN upon achieving complete remission (CR). The other 3 patients did not undergo IC due to molecular recurrence and a low tumor load, and they directly entered the consolidation and maintenance treatment with HMA ± VEN. The median number of HMA cycles was 4, ranging from 2 to 6 cycles. Among these patients, 7 received azacitidine at a dose of 75mg/m^2^/day through subcutaneous injection for 7 days, while 14 received decitabine at a dosage of 10mg/m^2^/day via intravenous infusions for 5 days (Regarding the dose of decitabine, the formulation was adjusted to 10mg/m^2^ based on the poor tolerance of marrow post HSCT). VEN was administered in 1-5 cycles, with a median of one cycle. Based on the research by Suresh K. Agarwal (19, 20), the dose of VEN was set at 100mg/day in combination with intravenous voriconazole to enhance the blood concentration of VEN. During the treatment, the blood concentration of VEN was regularly monitored to ensure its therapeutic efficacy. Each VEN cycle lasted for 2-3 weeks, followed by a 2-week interval before starting the next cycle.

In our patient cohort, not all donors consented to provide stem cells for a second time, patients who were eligible to receive donor cells again received DLI treatment concurrently. For those who were unable to receive additional donor cells, treatment was limited to IC or IC+HMA ± VEN only. Thirteen patients received DLI in the IC group, and 18 patients received DLI in the IC+HMA ± VEN group. The MNCs ranged from 1.02×10^8^/kg to 14.32×10^8^/kg with a median of 4.765×10^8^/kg in IC group; in the IC+HMAs ± VEN group, the MNCs range from 1×10^8^/kg to 12.98×10^8^/kg, with a median of 3.2×10^8^/kg.

### 2.3 Outcome measures

The response evaluation in this study adhered to international standards. Efficacy was assessed based on the rates of CR, CRi (CR with incomplete blood count recovery), PR (partial remission), and OS (overall survival) time. OS referred to the duration between relapse and the last follow-up or death from any cause. Disease-free survival (DFS) is the time from the initiation of randomization (or treatment commencement in a single-arm trial) to disease recurrence or death from any cause. Hematologic relapse occurred when the bone marrow blast count exceeded 5%. Molecular relapse was defined as a relapse or an increase in the proportion of initial disease-specific molecular markers. Alternatively, it included the loss of complete donor chimerism in the bone marrow or peripheral blood, as monitored by STR-PCR. Minimal residual disease (MRD) was monitored using established methods in accordance with institutional standards (17, 17). In our study, flow cytometry methods (FCM), often involving markers such as CD34 and CD117, were used. Certain molecular mutations and fusion genes, such as *RUNX1-RUNX1T1*, *CBFβ-MYH11*, *PML-RARα*, and *NPM1*, serve as reliable indicators for disease recurrence. These can be assessed as MRD evaluation markers via RT-PCR. Moreover, a substantial proportion of AML patients exhibit elevated expression of *WT1*, with a commonly established threshold set at 0.6%. This increased expression of *WT1* can also serve as an MRD indicator.

Adverse events were assessed and graded based on the National Cancer Institute’s Adverse Event Criteria (15). Bone marrow suppression was identified by a neutrophil count lower than 0.5×10^9^/L (agranulocytosis) and/or reductions in platelet and hemoglobin levels. For the patients in this study, an adverse event was considered to have occurred whenever an agranulocytosis (greater than or equal to one) occurred after receiving the treatment. In case of bone marrow suppression, the subsequent chemotherapy cycle would be delayed, and supportive treatments, such as component transfusions and anti-infection measures, would be provided. All medical records were tracked through the hospital database or via phone follow-ups. The median follow-up time was 8.5 months, ranging from 0.5 to 74 months.

### 2.4 Statistical analysis

The analysis of time-to-event endpoints, including overall survival (OS) and disease-free survival (DFS), utilized Kaplan-Meier curves. Stratification of relevant factors was carried out using log-rank tests, although this method may produce biased estimates when competing events are present, thus highlighting the need for competing risk models. In our study, non-recurrent deaths, such as those due to infections, were treated as competing events. To address this, we applied a competing risk model and performed both univariable and multivariable analyses alongside survival analysis. Statistical analysis was performed using R programming software. Mean ± standard deviation (X ± SD) values were utilized for normally distributed measurement data, while median values were reported for skewed data. Intergroup comparisons were conducted using the log-rank test. The chi-square test or Fisher’s exact test was employed for the comparison of categorical data. A two-sided P value less than 0.05 was considered statistically significant.

## 3 Results

### 3.1 Baseline characteristics

A retrospective review was conducted on 42 patients who experienced relapse after undergoing allogeneic hematopoietic stem cell transplantation (allo-HSCT) at our center between April 2012 and March 2022. Among these patients, 21 received HMAs treatment, and within the HMAs group, 13 out of 21 (62%) were male. Among the 21 patients, 11 were diagnosed with AML, 4 had secondary AML transformed from MDS, and 6 had MDS. Sixteen had intermediate/high risk molecular characteristics. Only 1 patient had a high leukocyte count at the onset. The median time to relapse from HSCT was 10 months, ranging from 2 to 64 months. In the HMAs group, 7 patients received treatment with VEN in combination with HMAs. Among these 7 patients, 6 were male and 1 was female, with a median age of 45 years. The median time to relapse was 4 months. Regarding diagnoses, 3 patients had Myelodysplastic Syndromes (MDS), 3 had Acute Myeloid Leukemia (AML), and 1 experienced progression from MDS to AML. Among the 21patients in the study, the median number of blasts was 26% (range: 9%-41%) for the HMAs group and 15% (range: 11%-50%) for the IC group. The p-value associated with these differences was calculated to be 0.874, indicating that there was no statistically significant variation in blast counts between the two treatment groups. For outcome comparison, an additional 21 patients who received chemotherapy only and experienced relapse post HSCT during the same period were selected. They were then matched to the HMAs group at a 1:1 ratio. The baseline characteristics of the patients are presented in **Table 1**, while the conditioning regimen and donor types were detailed in **Table 2**.

**Table 1:** Baseline characteristics.

Characteristic	Treatment		p-value
	HMAs, N = 21^1^	IC, N = 21^1^	
Age	41 (31, 51)	42 (27, 46)	0.435^2^
<20	3	5	
20-29	0	1	
30-39	7	4	
40-49	5	9	
≥50	6	2	
Gender			>0.999^4^
Female	8 (38.1%)	8 (38.1%)	
Male	13 (61.9%)	13 (61.9%)	
MDS	6 (28.6%)	0 (0.0%)	0.021^3^
AML	11 (52.4%)	16 (76.2%)	0.107^4^
t-AML	4 (19%)	5 (23.8%)	
WBC>100*10^9/L at diagnosis (%)	1 (4.8%)	6 (28.6%)	0.093^3^
DLI	18 (85.7%)	13 (61.9%)	0.079^4^
Extramedullary relapse	5 (24)	3 (14)	0.259
Time from HSCT to relapse (months)	7 (1.5-66)	10 (2-64)	0.538
Blast at relapse (%)	26 (9, 41)	15 (11, 50)	0.874^2^
Molecular characteristics classified as intermediate/high risk^5^ (%)	19 (90.5%)	18 (85.7%)	>0.999^3^
Donor Type			0.793^3^
Haplo-HSCT	14 (66.7%)	13 (61.9%)	
MSD-HSCT	5 (23.8%)	7 (33.3%)	
MUD-HSCT	2 (9.5%)	1 (4.8%)	

^1^Median (IQR); n (%).

^2^Wilcoxon rank sum test.

^3^Fisher’s exact test.

^4^Pearson’s Chi-squared test.

^5^Molecular characteristics with poor prognosis are classified according to the 2022 ELN recommendations.

Red colour indicates values less than 0.05.

**Table 2:** The transplant pre-treatment pr ogram and donor type.

	IC	IC+HMA ± VEN
Conditioning regimen		
Bu/Flu	13 (61.9)	6 (28.6)
Bu/Cy	3 (14.3)	1 (4.8)
D+Bu/Flu	4 (19.0)	12 (57.1)
D+Bu/Cy	1 (4.8)	2 (9.5)
Donor type		
MSD-HSCT	7 (33.3)	5 (23.8)
Haplo-HSCT	13 (61.9)	14 (66.7)
MUD-HSCT	1 (4.8)	2 (9.5)

Bu/Cy, Busulfan/Cyclophosphamide; Bu/Flu, Busulfan/Fludarabine; D, decitabine; MSD, HLA-matched sibling donors; Haplo-HSCT, HLA half matched sibling donor; MUD, HLA compatible unrelated donors.

### 3.2 ORR, OS and DFS

**Figure 1:**
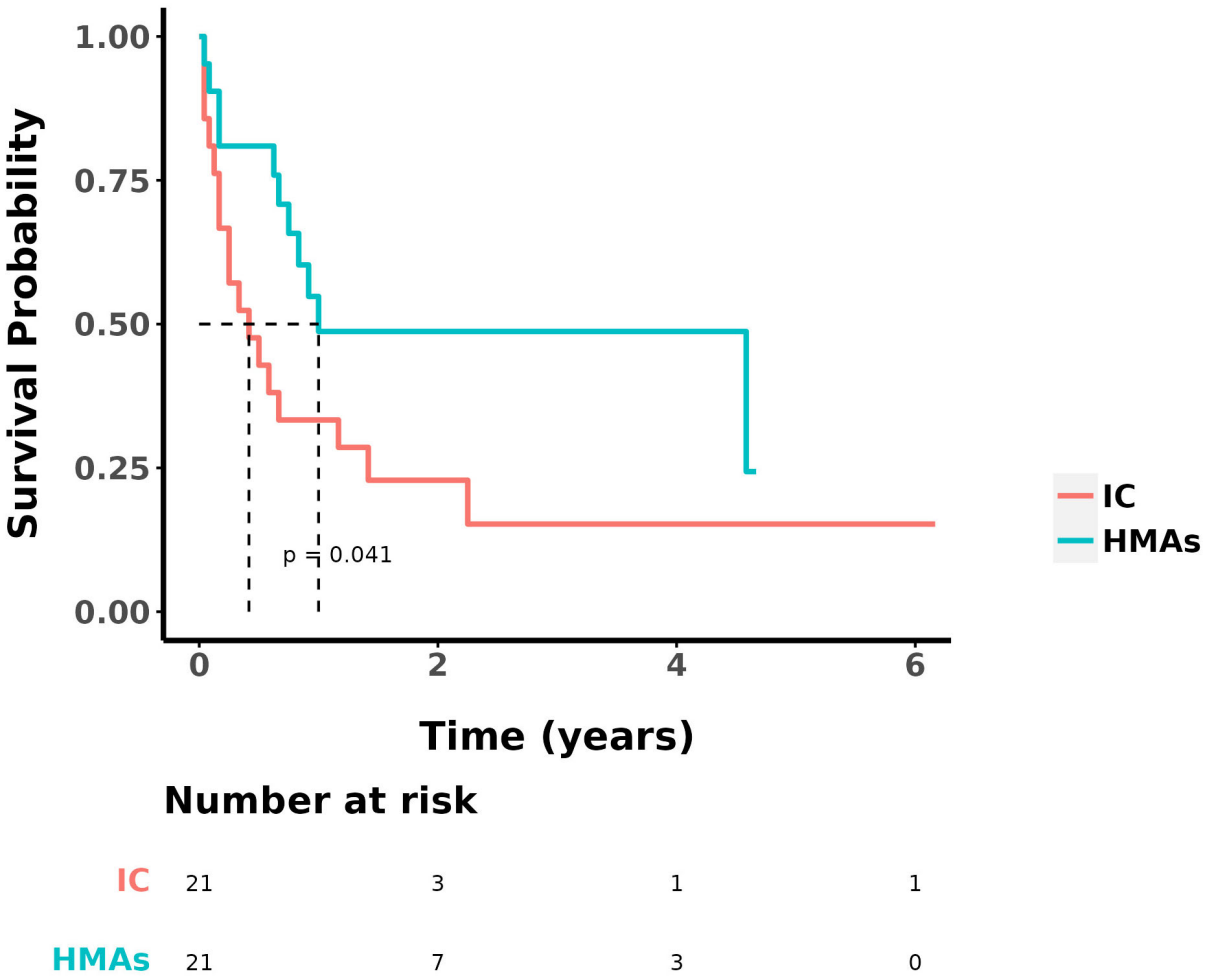
OS in IC group and IC+HMAs ± VEN group.

**Figure 2:**
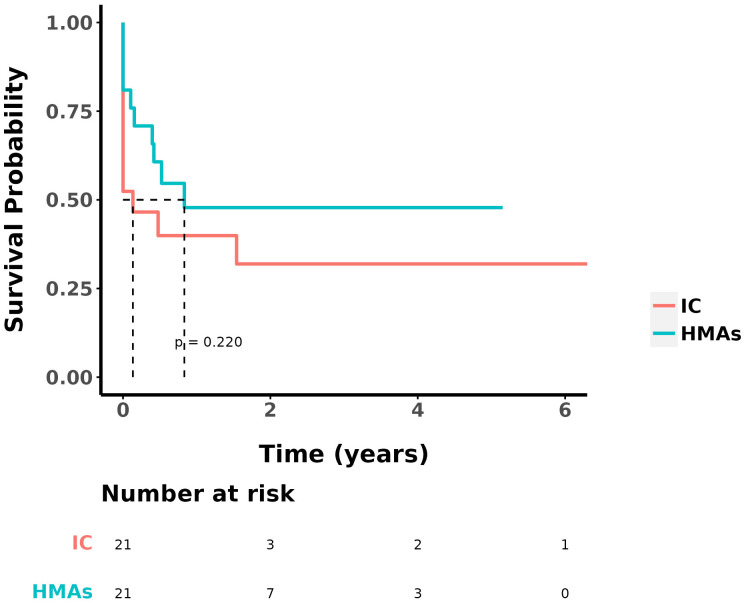
DFS in IC group and IC+HMAs ± VEN group.

At the study’s conclusion, the median OS was 9 ± 2.153 months, with a 1-year OS rate of 41.5%. The ORR in the IC group and the IC+HMA ± VEN group, encompassing complete remission (CR), CR with incomplete blood count recovery (CRi), CR with minimal residual disease-negative partial hematologic recovery (CRh), and partial remission (PR), was 52.38% (11/21) and 76.19% (16/21) respectively. No significant difference was observed in the ORRs between the two groups (P=0.107). The 1-year overall survival (OS) rate was 33.3% in the IC group and 48.7% in the IC+HMA ± VEN group. The median OS for the IC group was 0.4 years, and for the IC+HMAs ± VEN group, it was 1 year, demonstrating a significant difference between the two groups (HR=2.207, P=0.041, χ2 = 4.016). Regarding the 1-year disease-free survival (DFS) rate, the IC group had a rate of 39.9%, and the IC+HMAs ± VEN group had a rate of 47.8%. The median DFS was 0.1 year in the IC group and 0.8 year in the IC+HMAs ± VEN group. Nevertheless, no significant statistical difference was found between the two groups regarding DFS (P=0.220). These results are depicted in **Figure 1** (OS curves) and **Figure 2** (DFS curves).

**Figure 3:**
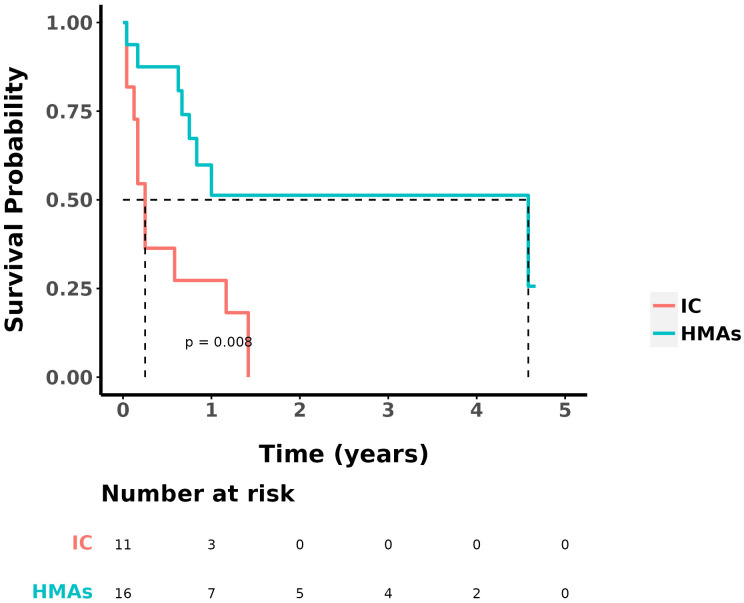
OS in IC group and IC+HMAs ± VEN group with intermediate/high-risk prognosis patients.

No statistically significant differences were found in OS and DFS between the two groups of patients who received DLI (P=0.100 and P=0.698, respectively). For all 42 patients, statistically analyzed by comparing OS, no statistically significant difference was seen in whether or not they received a DLI. Survival analysis was performed on patients with intermediate/high-risk cytogenetics in both groups. The median OS for the group treated with IC was 0.3 year, whereas it was 4.6 years for the group treated with IC+HMAs ± VEN. The 1-year OS rates were 27.3% in the IC group and 51.3% in the IC+HMA ± VEN group, respectively. A significant statistical difference was observed between the two groups (P=0.008), as illustrated in **Figure 3**.

**Figure 4:**
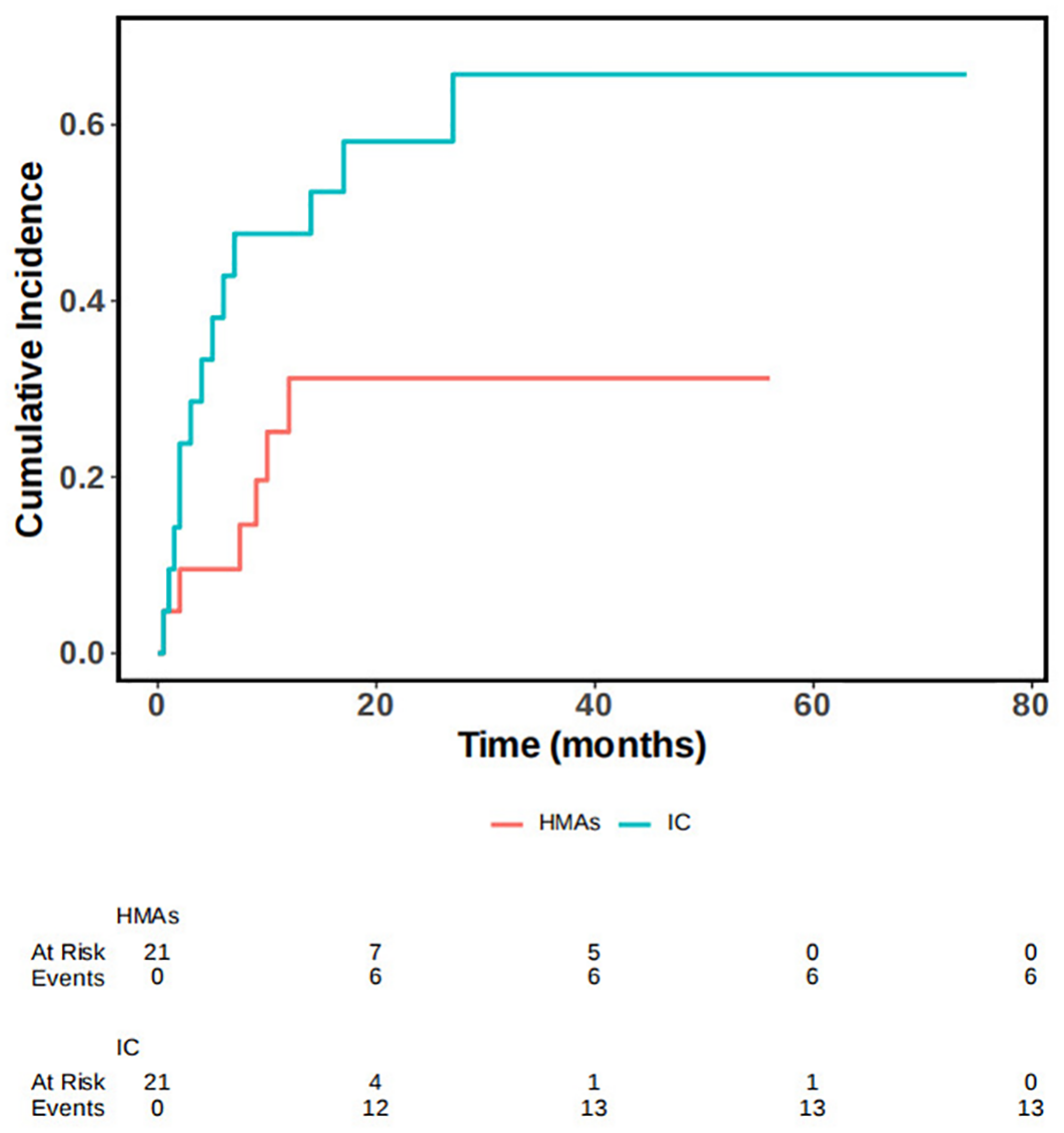
OS in IC group and IC+HMAs ± VEN group under the competitive risk model.

In our study, we considered non-recurrent deaths, such as those resulting from infections, as competing events. We conducted a competing risk model, performing single and multivariable analysis, as well as survival analysis. Statistical analyses of cumulative recurrence rates were also performed. In our multivariable analyses, we identified that the recurrence distance to transplantation time and the number of MNCs for DLI were statistically significant predictors of the outcome, while controlling for competing events. Death resulted from relapse was considered the primary event of interest, with non-relapse death as the competing event. We observed a statistically significant difference in the occurrence of the outcome event across different treatment modalities, while controlling for time to competition. Additionally, there was a statistical difference in the cumulative incidence function (CIF). Statistically significant differences in OS between the two groups were also seen when controlling for competing events (P=0.028), the survival curve was illustrated in **Figure 4**.

### 3.3 Secondary relapse

**Figure 5:**
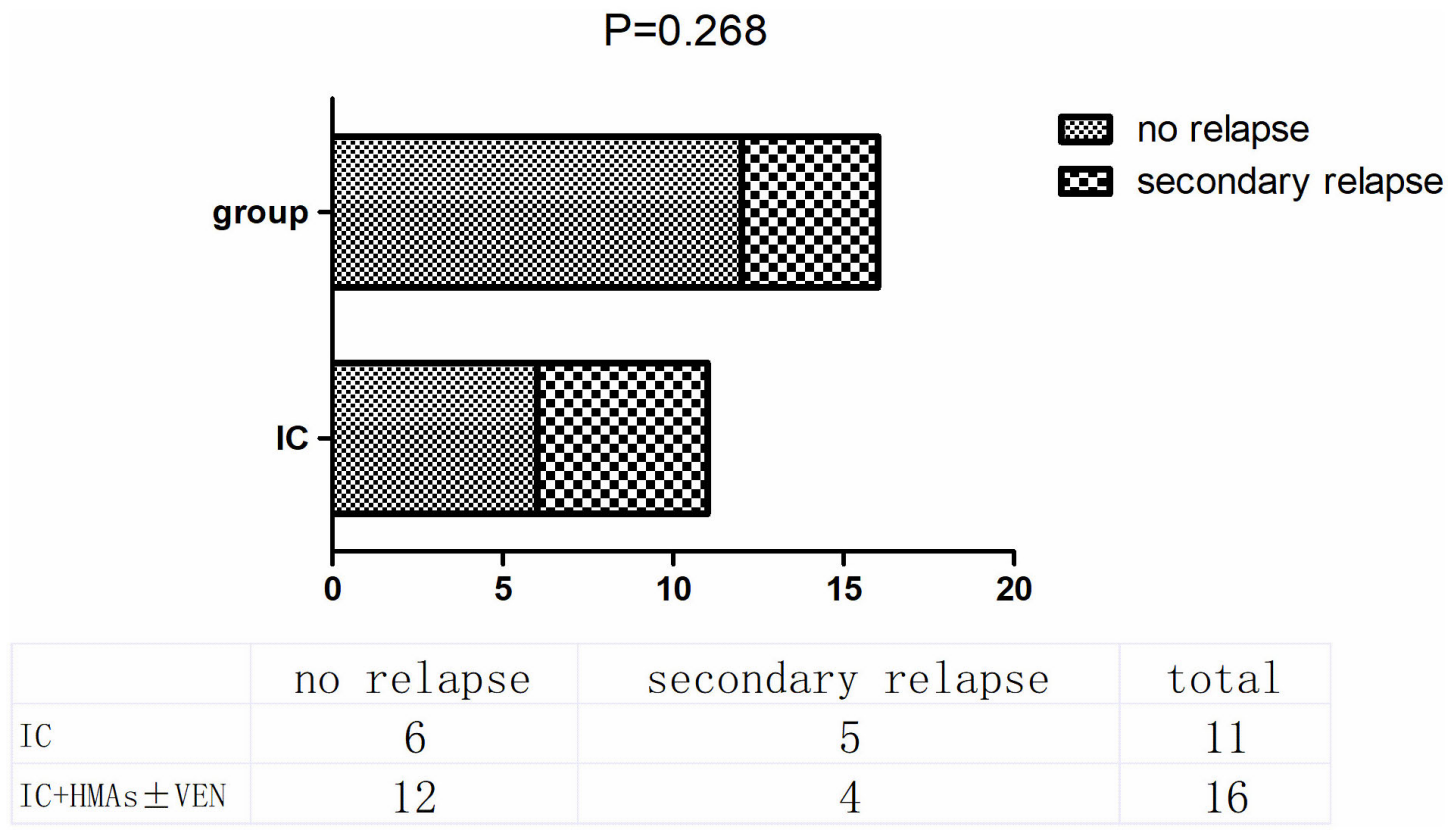
secondary relapse in IC group and IC+HMAs ± VEN group.

In the IC cohort, 11 patients responded to chemotherapy, including CR, CRi, CRh, and PR. At the conclusion of this retrospective study, 5 out of these 11 patients experienced secondary relapse, resulting in a relapse rate of 45.45%. In contrast, in the IC+HMAs ± VEN cohort, 16 achieved remission, and 4 experienced secondary relapse (25%). No significant difference was observed in the secondary relapse rate between the two cohorts (P=0.268), as illustrated in **Figure 5**.

### 3.4 AEs analysis

Adverse events in this study encompassed various infections (bacteria, fungi, CMV, EBV, etc.), bone marrow suppression, GvHD, and tumor lysis syndrome (TLS). In the IC group, 9 patients experienced grade II-IV GvHD, while 10 experienced grade II-IV GvHD in the IC+HMAs ± VEN group. No statistically significant difference was observed between the two groups (P=0.96). In the IC group, 16 patients experienced infections (bacteria, fungi, viruses were included), while 14 got infected in the IC+HMAs ± VEN group. No statistically significant difference was found between the two groups (P=0.495). Agranulocytosis occurred in 13 patients in the IC group, and 10 in the IC+HMA ± VEN group experienced agranulocytosis. No statistically significant difference was found between the two groups (P=0.352). The current grading system for myelosuppression after chemotherapy follows the World Health Organization criteria for grading acute and subacute toxic reactions to anticancer drugs. The most common adverse events were granulocyte deficiency, liver function abnormalities, and infection. No statistically significant difference was found (P=0.549). These findings are presented in **Table 3**.

**Table 3:** Adverse events in the two cohorts.

	IC (n=21)	HMA (n=21)	P
Infection^2^ (%)	16 (76.2)	14 (66.7)	0.595
II-IV° GvHD (%)	9 (42.9)	10 (47.6)	0.96
Agranulocytosis (%)	13 (61.9)	10 (47.6)	0.352
Grade 3/4 AEs	19 (90.5)	20 (95.2)	0.549

GVHD, graft versus host disease; ^2^: bacterium, fungus, CMV (cytomegalovirus), EBV (Epstein-Barr virus) infection were all included.

### 3.5 GvHD post DLI

**Figure 6:**
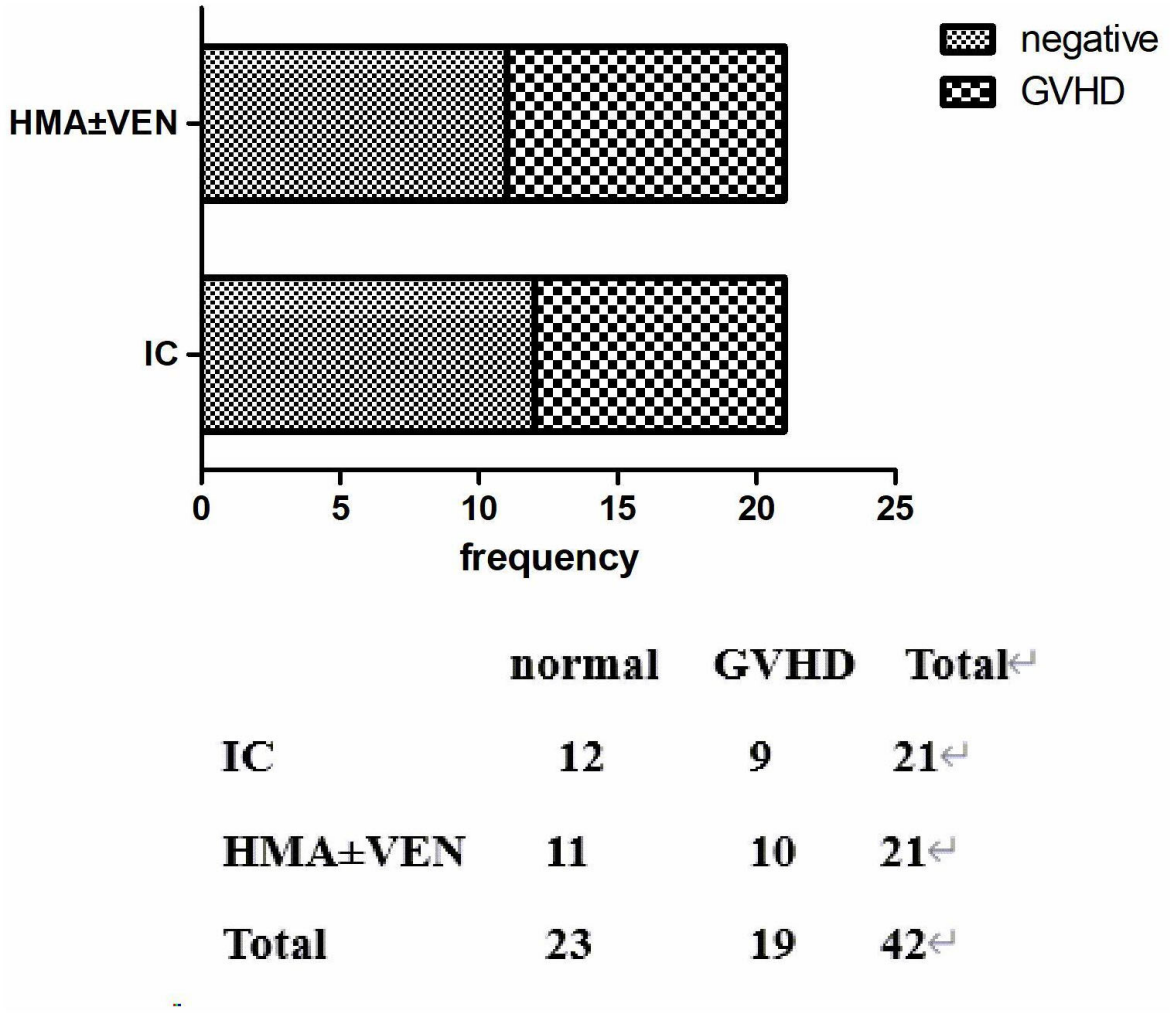
aGvHD in two groups after DLI.

In our study, patients could undergo pre-DLI or pro-DLI in the case of a molecular biology relapse or a hematological relapse. In the IC group, 17 patients received DLI as treatment, while 18 in the HMAs group received DLI. Among these patients, 5 out of 17 suffered from grade II-IV acute GvHD in the IC group, whereas 8 out of 10 experienced acute GvHD in the HMAs group. No significant difference was found between the two groups (P=0.4887), as illustrated in **Figure 6**.

In our multivariable analyses, we found that the time between relapse and transplantation, as well as the MNCs infused during DLI, were significantly associated with outcomes (P = 0.007), as summarized in **Table 4**.

**Table 4:** The Univariable and Multivariable competitive risk analysis.

Characteristic	Univariable					Multivariable				
	N	Event N	HR^1^	95% CI^1^	p-value	N	Event N	HR^1^	95% CI^1^	p-value
age	42	19	0.98	0.95, 1.01	0.170	25	10	1.02	0.96, 1.07	0.570
DLI										
Yes	31	14	—	—						
No	11	5	0.91	0.35, 2.34	0.840					
Rtime	42	19	0.92	0.85, 0.99	0.031	25	10	0.47	0.31, 0.71	<0.001
treatment										
HMAs	21	6	—	—		15	5	—	—	
IC	21	13	2.70	1.05, 6.95	0.039	10	5	0.32	0.08, 1.30	0.110
DLI-MNC	25	10	1.07	0.93, 1.24	0.330	25	10	1.22	1.06, 1.42	0.007

^1^HR, Hazard Ratio; CI, Confidence Interval.

converged = 1.00; Log-likelihood = -18.4; No. Obs. = 25; df = 4; Statistic = 21.9.

Red colour indicates values less than 0.05.

### 3.6 Drug concentration monitoring

Patients receiving treatment with VEN started at a dose of 100 mg/day, utilizing triazole antifungals to attain sufficient blood concentration. Patients treated with the combination of HMAs and VEN exhibited a median blood concentration of 1687.7 ug/L (22.1-2979.0) in the initial treatment cycle. Among these patients, four achieved negative MRD and were classified as having achieved CR, while 2 showed no response, yielding an ORR of 66.7% (4/6). The remaining two patients continued HMAs+VEN therapy with doses adjusted based on blood drug concentration, reducing the dose when the concentration exceeded 3000 ug/L or if adverse reactions occurred. One patient experienced acute kidney injury (AKI) and severe gastrointestinal reactions when the VEN concentration reached 2256.93 ug/L. However, symptoms improved after reducing the VEN dose.

## 4 Discussion

AML and MDS are heterogeneous myeloid malignancies, and chemotherapy has traditionally been the primary treatment. However, the remission rate and long-term survival rate for chemotherapy are both below 30%. Advances in allogeneic hematopoietic stem cell transplantation technology provide the potential for long-term survival in AML/MDS patients. Over the past decade, transplantation-related mortality has significantly decreased. However, post-transplantation relapse remains a major cause of reduced survival rates, and the treatment options for post-transplantation relapse are limited. Re-administering chemotherapy after relapse may result in short-term remission for some patients, but the relapse rate is exceedingly high. Additionally, the risk associated with secondary transplantation is substantial, and the majority of patients are ineligible for secondary transplantation following disease relapse. Recently, new treatments have been explored, including novel agents like BCL2 inhibitors, demethylating agents, and idasanutlin plus cytarabine, among others (21–27). These advancements provide hope for enhanced therapeutic strategies and improved outcomes for patients with AML/MDS experiencing relapse post allo-HSCT.

Relapse post hematopoietic stem cell transplantation (HSCT) may result from cancer cells evading the effects of chemotherapy or the immune system. Therefore, infusing CD3+ T cells from the donor could augment the donor-versus-leukemia effect, addressing the primary disease (22). In a retrospective study conducted by the European Society for Blood and Marrow Transplantation (EBMT)-Acute Leukemia Working Party (ALWP), involving 399 patients, 171 received DLI. The study revealed a statistically significant difference in the 2-year OS rate between individuals who received DLI and those who underwent other treatments (21 ± 3% vs. 9 ± 2%, P<0.001) (4). However, the most prevalent adverse effect was GvHD, which can be life-threatening (28). Current treatment options are constrained by efficacy and adverse effects. There is an urgent need to discover new treatments that could reduce the incidence of adverse reactions while enhancing efficacy.

Treating relapsed AML/MDS patients post-HSCT with HMAs alone has resulted in unstable therapeutic effects, with response rates ranging from 0% to 75% (29). Consequently, we have improved the treatment approach by incorporating single-agent maintenance therapy with HMAs after 1-2 cycles of chemotherapy to attain CR in these patients. In order to ensure the rigor of the study, we compared the number of patients who reached CR after 1-2 cycles of chemotherapy in both groups, in the IC group, 17 patients achieved CR in bone marrow morphology after 1-2 cycles of chemotherapy, while in the HMAs group, 18 patients with hematological relapse achieved CR after 1-2 cycles of chemotherapy as suggested by the bone puncture, the chi-square analyses were performed in the two groups, and the Fisher’s precision test did not show a statistically significant difference (P=0.10), no statistically significant difference was seen, while the final comparison of survival data between the IC and HMAs ± VEN groups was statistically different, so we can conclude that the efficacy of HMA ± VEN for maintenance therapy is superior to the maintenance of IC.

In this study, we observed that treatment with IC+HMAs ± VEN resulted in improved OS compared to the IC group (P=0.041). Additionally, the IC+HMAs ± VEN group exhibited superior outcomes in patients with intermediate or high-risk cytogenetics (P=0.008). Moreover, among the 7 patients in the HMAs group treated with VEN, no statistically significant difference in OS was found when compared to patients who received HMAs alone (P=0.183). This contrasts with a study by DiNardo (30) which demonstrated that AZA+VEN treatment significantly improved median survival (14.7 months vs. 9.6 months; P<0.001) and CR rates (66.4% vs. 28.3%; P<0.001) in patients with AML, as compared to AZA with a placebo, based on standard therapy (30). However, the lack of significance in our study may be attributed to its small sample size. Regarding the secondary relapse rate, we observed a reduction in the IC+HMA ± VEN cohort compared to the IC cohort; however, the difference was not statistically significant. To enhance the rigor of our results, we conducted a competing risks analysis, accounting for non-recurrent deaths (such as deaths unrelated to disease recurrence) as competing events. This analysis revealed statistically significant differences in overall survival (OS) between the two patient groups. Both univariate and multivariate analyses supported these findings, further reinforcing our conclusion that the use of demethylating agents, with or without venetoclax, significantly improves survival in patients with recurrent medullary tumors following transplantation. This comprehensive approach strengthens the validity of our findings. This lack of significance may be attributed to the limitations in the sample size for this study.

In this study, two types of HMAs were utilized. Consistent with our results, a study conducted by Yun-Gyoo Lee found no significant difference in the effectiveness of these two drugs when comparing OS and event-free survival (EFS) (P=0.85, 0.96) (31, 32). This suggests that both HMAs may yield similar therapeutic outcomes for patients, indicating that either of the two agents could be considered in treatment planning without substantially compromising therapy effectiveness.

According to Agarwal SK’s study, the combination of VEN with triazole antifungals, such as ketoconazole and posaconazole, improves both the concentration-time curve (AUC∞) and the half-time (t_1/2_) (19, 20, 33). This enhancement permits a reduction in the VEN dose while maintaining efficacy. In a previous retrospective study, VEN was administered to patients with AML and high-risk MDS who were unsuitable for intensive chemotherapy due to neutropenia. We analyzed VEN drug concentrations and correlated them with therapeutic responses and adverse events by monitoring exposure levels of VEN and posaconazole/voriconazole. Our findings revealed that a trough concentration (Cmin) within the therapeutic range was significantly associated with a higher rate of minimal residual disease (MRD) negativity (90.91% vs. 33.33%, p=0.028). In contrast, abnormal peak concentrations did not significantly impact MRD negativity (77.78% vs. 63.50%, p=0.620) (34). Given the poor bone marrow tolerance in post-transplant patients, we initiated VEN treatment at 100 mg/day, with continuous monitoring of drug concentrations. In conclusion, regular monitoring of hematologic and biochemical parameters is essential to ensure effective treatment with VEN, and dosage adjustments should be made as necessary.

Additionally, our multivariable analysis revealed that the number of mononuclear cells (MNCs) infused during DLI had a statistically significant impact on patient survival. This finding highlights the critical role of MNC dosage as a potential prognostic factor for patients undergoing DLI, underscoring the importance of optimizing MNC dosage to improve treatment outcomes.

The primary adverse reactions in this study included bone marrow suppression and infection. Comparing adverse reactions between IC+HMAs ± VEN therapy and chemotherapy revealed no significant difference in the incidence of II-IV° GvHD and agranulocytosis.

Adverse events, encompassing acute and chronic GvHD, infections (CMV, EBV, fungus, bacteria included), and the incidence of agranulocytosis, showed no significant differences between the two groups (**Table 2**, 76.2% vs. 66.7%, P = 0.595; 42.9% vs. 47.6%, P = 0.96; 61.9% vs. 47.6%, P = 0.352).

It is essential to note that the conclusions drawn from this study are limited by its small sample size and potential biases. However, the study’s findings suggest that treatment with HMAs can be an effective therapeutic option for relapsed acute myeloid leukemia/myelodysplastic syndromes (AML/MDS) cases post allo-HSCT, especially for cases with intermediate/high-risk cytogenetics. Adding VEN to HMAs therapy may further enhance prognosis and extend survival time. For patient safety and optimal treatment outcomes, meticulous monitoring of drug concentrations, blood parameters, and liver and kidney function during treatment is crucial. This approach can prevent adverse reactions and ensure proper dosing of VEN.

## 5 Conclusion

In conclusion, this retrospective cohort study provides evidence that hypomethylating agents represent an effective and safe treatment option for relapsed myeloid malignancies following allo-HSCT, especially for cases with intermediate/high-risk cytogenetics. The inclusion of VEN in HMA therapy may enhance prognosis and extend survival time. Nonetheless, it is crucial to recognize the limitations of this study, including its small scale, single-center, and retrospective design. To validate these findings rigorously, a prospective, multicenter randomized controlled trial is essential.

## Data Availability

The data analyzed in this study is subject to the following licenses/restrictions: Data will be available on request from the authors. Requests to access these datasets should be directed to YL, drliyuan75@163.com.
